# Measurement of lipocalin-2 and syndecan-4 levels to differentiate bacterial from viral infection in children with community-acquired pneumonia

**DOI:** 10.1186/s12890-016-0267-4

**Published:** 2016-07-20

**Authors:** Susanna Esposito, Sonia Bianchini, Monia Gambino, Barbara Madini, Giada Di Pietro, Giulia Umbrello, Maria Lory Presicce, Luca Ruggiero, Leonardo Terranova, Nicola Principi

**Affiliations:** Pediatric Highly Intensive Care Unit, Department of Pathophysiology and Transplantation, Università degli Studi di Milano, Fondazione IRCCS Ca’ Granda Ospedale Maggiore Policlinico, Via Commenda 9, 20122 Milan, Italy

**Keywords:** Biomarkers, Community-acquired pneumonia, C reactive protein, Lipocalin 2, Syndecan 4, White blood cell count

## Abstract

**Background:**

In this study, we evaluated the lipocalin-2 (LIP2) and syndecan-4 (SYN4) levels in children who were hospitalized for radiologically confirmed CAP in order to differentiate bacterial from viral infection. The results regarding the LIP2 and SYN4 diagnostic outcomes were compared with the white blood cell (WBC) count and C reactive protein (CRP) levels.

**Methods:**

A total of 110 children <14 years old who were hospitalized for radiologically confirmed CAP were enrolled. Serum samples were obtained upon admission and on day 5 to measure the levels of LIP2, SYN4, and CRP as well as the WBC. Polymerase chain reaction of the respiratory secretions and tests on blood samples were performed to detect respiratory viruses, *Streptococcus pneumoniae*, and *Mycoplasma pneumoniae*.

**Results:**

CAP was considered to be due to a probable bacterial infection in 74 children (67.3 %) and due to a probable viral infection in 16 children (14.5 %). Overall, 84 children (76.4 %) were diagnosed with severe CAP. The mean values of the WBC count and the LIP2 and SYN4 levels did not differ among the probable bacterial, probable viral, and undetermined cases. However, the CRP serum concentrations were significantly higher in children with probable bacterial CAP than in those with probable viral disease (32.2 ± 55.5 mg/L vs 9.4 ± 17.0 mg/L, *p* < 0.05). The WBC count was the best predictor of severe CAP, but the differences among the studied variables were marginal. The WBC count was significantly lower on day 5 in children with probable bacterial CAP (*p* < 0.01) and in those with an undetermined etiology (*p* < 0.01). The CRP and LIP2 levels were significantly lower 5 days after enrollment in all of the studied groups, independent of the supposed etiology of CAP (*p* < 0.01 for all comparisons). No statistically significant variation was observed for SYN4.

**Conclusions:**

Measuring the LIP2 and SYN4 levels does not appear to solve the problem of the poor reliability of routine laboratory tests in defining the etiology and severity of pediatric CAP. Currently, the CRP levels and WBC, when combined with evaluation of clinical data, can be used to limit the overuse of antibiotics as much as possible and to provide the best treatment to the patient.

## Background

Community-acquired pneumonia (CAP) is one of the leading causes of morbidity and mortality in young children worldwide [1]. Viruses and bacteria, either alone or in combination, are the primary causes of CAP [2, 3]. Early detection of bacterial cases is essential to guide clinical management and avoid prolonged hospitalization, complications and the risk of death [4]. Furthermore, differentiation of viral CAP from bacterial CAP is the basis for the rational administration of antibiotics, the reduction in the use of antibiotics and the consequent reduction of the emergence of bacterial resistance and drug-related adverse events [5, 6]. Unfortunately, these goals are difficult to attain, particularly in children in whom the collection of respiratory samples is either difficult or impossible [6]. The clinical signs, symptoms and radiological findings are frequently similar in viral and bacterial diseases. Moreover, in most cases, the results of routine laboratory tests, including white blood cell (WBC) count and serum C-reactive protein (CRP) levels, tend to overlap, making differentiation between viral and bacterial infection impossible [7, 8]. These limitations explain why several attempts to find more effective markers of bacterial etiology and the severity of CAP have been made in recent years.

Recently, it has been suggested that lipocalin-2 (LIP2), also known as neutrophil gelatinase-associated lipocalin (NGAL), and syndecan-4 (SYN4) could be useful to diagnose bacterial and severe CAP [9–12]. LIP2 is a protein that is stored in the granules of human neutrophils and has been described as a component of the innate immune system and the acute phase response to infection [13]. This protein plays a relevant role in innate defense primarily by interfering with bacterial iron uptake [14]. LIP2-deficient animals exhibit higher mortality rates compared to control animals when they are challenged with pathogenic bacteria [14, 15]. Moreover, LIP2 is upregulated in inflammatory states [15], and its expression is strongly increased in bronchial epithelial cells and alveolar type 2 pneumocytes of animals exposed to bacterial pathogens [16]. Viral infections did not seem to influence LIP2 levels [13–15].

SYN4 is a heparin sulfate proteoglycan that is expressed on the surface of a variety of cells, including macrophages, epithelial cells, endothelial cells, and fibroblasts [17, 18]. SYN4 binds to several cytokines, chemokines, and growth factors and mediates their biological activity. SYN4 expression is rapidly increased in response to bacterial infection and not to viral infection [17, 18]. SYN4-deficient animals have an increased risk of negative evolution when exposed to bacteria [19]. Finally, pre-treatment of lung epithelial cells with SYN4 was observed to inhibit the inflammatory properties of bacterial components such as lipopolysaccharides and proinflammatory cytokines [11].

Data collected in humans regarding these proteins are scarce, and the real role of LIP2 and SYN4 as biomarkers for defining the etiology and severity of CAP is poorly defined. No data on these new biomarkers are available in children and due to their characteristics they appear attractive in the possibility to differentiate bacterial from viral CAP. However, it is unclear whether the inclusion of routine laboratory tests detecting these proteins could be useful in routine clinical practice. This study was planned to evaluate the LIP2 and SYN4 levels in a group of children who were hospitalized in pediatric ward for radiologically confirmed CAP in order to differentiate bacterial from viral infection. The results regarding the diagnostic performances of LIP2 and SYN4 were compared with those of the WBC count and serum CRP levels.

## Methods

### Patient enrolment

This study was carried out at the Pediatric Highly Intensive Care Unit of the University of Milan, Fondazione IRCCS Ca’ Granda Ospedale Maggiore Policlinico, between November 1, 2014, and April 30, 2015, and was approved by the Ethics Committee of the Fondazione IRCCS Ca’ Granda Ospedale Maggiore Policlinico in Milan, Italy. Written informed consent was obtained from either the parent(s) or legal guardian(s) of each study participant, and the children who were aged >8 years signed their written assent.

Only otherwise healthy children below 14 years of age with clinical signs such as tachypnea and abnormal breath sounds that suggested CAP and who required hospitalization were recruited. The CAP diagnoses were confirmed by chest radiography, as evaluated by an independent expert radiologist, who classified the findings as alveolar pneumonia, non-alveolar pneumonia, or no pneumonia in accordance with the World Health Organization criteria for the standardized interpretation of pediatric chest radiographs for a diagnosis of pneumonia [20]. Alveolar pneumonia was defined as dense opacity appearing as fluffy consolidation of either part or all of a lobe, or an entire lung, often containing air on bronchography and occasionally associated with pleural effusion.

Disease severity was established following the criteria indicated for children by the British Thoracic Society [21]. In particular, the features of severe disease in an infant were as follows: oxygen saturation <92 %; cyanosis; respiratory rate >70 breaths/min; significant tachycardia for level of fever; prolonged central capillary refill time >2 s; difficulty breathing; intermittent apnea or grunting; and not feeding. The features of severe disease in an older child included the following: oxygen saturation <92 %; cyanosis; respiratory rate >50 breaths/min; significant tachycardia for level of fever; prolonged central capillary refill time >2 s; difficulty in breathing; grunting; and signs of dehydration.

After enrollment, the demographics, clinical history and clinical disease characteristics of each child were recorded. Moreover, a blood sample was collected for routine laboratory testing, including WBC count, serum CRP levels, blood culture and LIP2 and SYN4 levels. The sample was divided into two aliquots; the first was sent to the central laboratory of the hospital for routine testing, including WBC count, serum CRP levels and blood culture, whereas the second aliquot was sent to a research laboratory to measure the serum LIP2 and SYN4 concentrations and to detect *Streptococcus pneumoniae* and *Mycoplasma pneumoniae* DNA. Moreover, a nasopharyngeal sample was obtained using a pernasal nylon flocked swab and stored in a tube of universal transport medium (Kit Cat. No. 360c, Copan Italia, Brescia, Italy) for virus, *S. pneumoniae*, and *M. pneumoniae* detection.

All of the hospitalized children were treated according to international guidelines [22]. The clinical data collected during hospitalization were recorded daily, and all of the enrolled children were re-evaluated after 5 days by means of interviews and clinical examinations performed by trained investigators using standardized questionnaires. A second blood sample was drawn during the visits on day 5 to determine the WBC count and the serum concentrations of CRP, LIP2, and SYN4.

### LIP2 and SYN4 determination

Freshly thawed aliquots of serum samples were used for LIP2 and SYN4, and their analyses were performed by two different commercial enzyme-linked immunosorbent assay (ELISA) kits: a human LIP2 ELISA kit (Biolegend, San Diego, CA, USA) and a human SYN4 ELISA Kit (CUSABIO Life Science Biotech Co., Hubei Province, China); both kits were used according to the manufacturer’s instructions. All of the serum samples were clear and non-hemolyzed. The average minimum detectable concentration of the human LIP2 kit was 16.4 ng/mL and the minimum detectable level of the human SYN4 kit was less than 0.10 ng/mL.

### Viral analyses

Viral RNA or DNA was extracted from the respiratory secretions by means of a NucliSENS EasyMAG automated extraction system (Biomeriéux, Craponne, France) and was then tested using the Luminex xTAG respiratory virus panel fast assay (Luminex Molecular Diagnostics Inc., Toronto, Canada) to detect influenza A virus (subtypes H1 or H3); influenza B virus; respiratory syncytial virus (RSV) A and B; parainfluenzavirus-1, 2, 3, and 4; adenovirus, human metapneumovirus; coronaviruses 229E, NL63, OC43, and HKU1; enterovirus/rhinovirus (RV) and human bocavirus [23–25] in accordance with the manufacturer’s instructions. The enterovirus/RV-positive samples were retested by means of a real-time polymerase chain reaction (PCR) assay using the iAg-Path-ID one-step real-time PCR kit (Applied Biosystems, Foster City, CA, USA), and the primers and probe sequences reported by Lu et al. [26] to identify the RV.

The remaining amounts of nucleic acid extracts were used for bacterial analyses.

### Identification of *Streptococcus pneumoniae* and *Mycoplasma pneumoniae*

In addition to blood culture (that included also *Staphylococcus aureus* culture), advanced methods for research of the two main pathogens (i.e., *S. pneumoniae* and *M. pneumoniae*) involved in the etiology of pediatric CAP were performed.

To identify the pneumococcal cases, the nasopharyngeal samples and the blood collected upon hospital admission were tested for the autolysin-A (*LytA*) and wzg (*cpsA*) genes of *S. pneumoniae* using real-time PCR as previously described [27]. Each sample was tested in triplicate and was considered positive if at least two of the three replicates were positive. To maximize sensitivity, no internal amplification control was used in the reaction, but an external control was used.

In the nasopharyngeal samples and the blood collected at admission, *M. pneumoniae* DNA was tested with a validated nested PCR as previously described [28].

### Identification of probable bacterial and viral infection

CAP was considered to be of probable bacterial origin under two criteria: 1) detection of any bacteria in blood culture or detection of *S. pneumoniae* or *M. pneumoniae* either in the blood or in a nasopharyngeal swab positive in absence of viral detection and 2) a chest radiograph suggesting alveolar CAP.

Probable viral CAP was diagnosed in presence of a nasopharyngeal swab positive for one or more respiratory viruses in absence of bacterial detection and in association with a chest radiograph leading to diagnosis of non-alveolar CAP.

Undetermined cases included those with both bacterial and viral pathogens or those with no pathogen detected.

### Statistical analysis

Descriptive statistics of the responses were generated. Continuous variables were presented as the mean values ± standard deviation (SD) and the categorical variables as numbers and percentages. For categorical data, comparisons between groups were performed using the contingency table analysis with either the *χ*^2^ or Fisher’s exact test as appropriate. Continuous data were analyzed using either a two-sided Student’s *t*-test after determining that the data were normally distributed (based on the Shapiro-Wilk statistic) or a two-sided Wilcoxon’s rank-sum test.

Diagnostic performances of the biomarkers were evaluated via receiver operating characteristic (ROC) curves and the area under the ROC curve (AUC). The best cutoff values for different biomarkers were obtained based on the highest sensitivity and specificity using the roctab function in STATA. Odds ratios (OR) and 95 % confidence intervals (CI) were calculated to measure the association between biomarkers and the following dependent variables: 1) probable bacterial etiology; 2) probable viral etiology; and 3) disease severity. ORs were obtained using unconditional multiple logistic regression and were adjusted for age, sex, ethnicity, education of parents, and smoking habits of parents.

All analyses were conducted using SAS version 9.2 (Cary, NC, USA) and STATA version 11.0 (StataCorp LP, College Station, TX, USA).

## Results

A total of 110 children (males, 61 [55.4 %]; mean age 4.8 ± 3.4 years) were enrolled. Their demographic, clinical, and laboratory characteristics are reported in Table 1. CAP was considered due to a probable bacterial infection in 74 children (67.3 %) and a probable viral infection in 16 children (14.5 %). In 20 cases (18.2 %), it was not possible to identify the probable etiology of the disease; these patients were defined as undetermined. Overall, 84 (76.4 %) children had severe CAP. The mean values of the WBC count and LIP2 and SYN4 levels did not differ between probable bacterial, probable viral, and undetermined cases. However, the serum CRP concentrations were significantly higher in children with probably bacterial CAP than in those with probable viral disease (32.2 ± 55.5 mg/L vs 9.4 ± 17.00 mg/L, *p* < 0.05).

**Table 1:** Demographic, clinical, and laboratory variables in 110 children hospitalized for radiologically confirmed community-acquired pneumonia according to their infection status

	All subjects	Probable bacterial	Probable viral	Undetermined
	*n* = 110	*n* = 74	*n* = 16	*n* = 20
Demographics and clinical presentation				
Males (%)	61 (55.4)	45 (60.8)*	5 (31.2)*	11 (55.0)
Mean age ± SD (years)	4.8 ± 3.4	5.1 ± 3.4	3.6 ± 2.8	4.9 ± 3.5
Caucasians (%)^a^	95 (88.8)	62 (87.3)	13 (81.2)	20 (100.0)
At least one parent smoked (%)^a^	28 (25.9)	19 (26.0)	5 (31.2)	4 (21.0)
Presence of fever” (%)	91 (82.7)	64 (86.5)	12 (75.0)	15 (75.0)
O_2_ therapy (%)^a^	26 (25.2)	14 (19.7)*	7 (50.0)*	5 (27.8)
Clinical findings (%)				
Rhonchi	12 (10.9)	5 (6.8)	3 (18.7)	4 (20.0)
Rales	92 (83.6)	63 (85.1)	14 (87.5)	15 (75.0)
Wheezing	21 (19.1)	11 (14.9)	3 (18.7)	7 (35.0)
Any of the above	96 (87.3)	64 (86.5)	15 (93.7)	17 (85.0)
Severe disease	84 (76.4)	59 (79.7)	12 (75.0)	13 (65.0)
Laboratory data				
WBC (cells/μL)^a^	15,741 ± 8,575	16,573 ± 8,969	13,674 ± 7338	14,358 ± 7,899
Neutrophils, %^a^	66.1 ± 19.3	68.3 ± 18.4	65.2 ± 17.8	59.2 ± 22.9
CRP, μg/dL^a^	25.5 ± 47.4	32.2 ± 55.5*	9.4 ± 17.0*	14.0 ± 19.4
LIP2, ng/mL	2,079 ± 1,606	2,158 ± 1,744	1,880 ± 1,015	1,946 ± 1,488
SYD4, ng/mL	5.08 ± 3.95	5.27 ± 4.10	4.31 ± 3.06	4.99 ± 4.14

*CRP* C-reactive protein, *LIP2* lipocalin-2, *SD* standard deviation, *SYD4* syndecan-4, *WBC* white blood cell count

*p < 0.05 between probable bacterial and probable viral infection groups. No other statistically significant differences between groups were observed

^a^Some missing values (two cases in probable bacterial CAP, two in probable viral CAP and two in undetermined CAP)

Table 2 summarizes microbiological findings in children classified as probable bacterial or probable viral infection. Blood culture was negative in all the cases. Probable bacterial infections were due to *S. pneumoniae* (59 cases) or to *M. pneumoniae* (15 cases). Probable viral infections were due to RSV (12 cases), metapneumovirus (2 cases) and influenza A (2 cases).

**Table 2:** Frequency of infections detected among children hospitalized for radiologically confirmed community-acquired pneumonia and probable bacterial or probable viral infection

Type of infection	*n* (%)
Probable bacterial	74
*Streptococcus pneumoniae* infection (swab only)	59/74 (79.7)
*Mycoplasma pneumoniae* infection (swab only)	15/74 (20.3)
Probable viral	16
Respiratory syncytial virus	12/16 (75.0)
Metapneumovirus	2/16 (12.5)
Influenza A	2/16 (12.5)

Table 3 shows the diagnostic performance of the four studied biomarkers to discriminate bacterial and viral infections and identify severe CAP according to biomarker cut-off with the highest sensitivity and specificity. As evidenced by the AUC, all of the biomarkers were insufficient to identify probable bacterial and probable viral cases. Regarding probable bacterial infections, CRP level was the best predictor. A serum CRP concentration ≥7.4 mg/L was observed to be associated with a sensitivity of 64.4 % and a specificity of 69.4 %, leading to a positive predictive value (PPV) of 81 % and a negative predictive value (NPV) of 49 %. The AUC was 0.65 (95 % CI 0.53-0.76). Both LIN2 and SYN4 had lower predictive ability. For both, the AUC was low (0.51, 95 % CI 0.40-0.63 for LIN2 and 0.54, 95 % CI 0.42-0.65 for SYN4). However, whereas the cutoff value of LIP2 (1,633 ng/mL) had limited sensitivity and specificity (58.1 % and 50.0 %, respectively), that of SYN4 (≥7.25 ng/mL) was shown to have very low sensitivity (31.1 %) but very high specificity (86.1 %), leading to a PPV of 82.1 % and to a NPV of 37.8 %. The AUC for the WBC count was 0.57 (95 % CI 0.46-0.69). The cutoff value of the WBC count (≥10,300) had a sensitivity of 74 %, a specificity of 38.9 %, a PPV of 71.1 %, and a NPV of 42.4 %.

**Table 3:** Diagnostic performance of lipocalin-2 and syndecan-4 biomarkers compared to white blood cell count and C reactive protein at time of enrollment in 110 children hospitalized for radiologically confirmed community-acquired pneumonia to predict infections and severity of disease, and odds ratio and 95 % confidence interval according to the biomarker cut-off with the highest sensitivity and specificity

	Biomarker cut-off value	Sensitivity	Specificity	PPV	NPV	AUC (95 % CI)	OR (95 % CI)***
*S. pneumoniae* infection (*n* = 59)							
LIP2	≥1,633	71.2 %	62.7 %	68.9 %	65.3 %	0.62 (0.51-0.73)	4.26 (1.76-10.3)
SYN4^a^	≤1.78	28.8 %	80.4 %	63.0 %	49.4 %	0.50 (0.39-0.61)	1.78 (0.62-5.15)
WBC	≥13,500	63.8 %	68.6 %	69.8 %	62.5 %	0.66 (0.55-0.76)	3.91 (1.50-10.2)
CRP	≥12.3	58.6 %	68.6 %	68.0 %	59.3 %	0.63 (0.52-0.74)	2.59 (1.03-6.54)
Probable bacterial infection (*n* = 74)							
LIP2	≥1,633	58.1 %	50.0 %	70.5 %	36.7 %	0.51 (0.40-0.63)	1.21 (0.49-2.99)
SYN44	≥7.25	31.1 %	86.1 %	82.1 %	37.8 %	0.54 (0.42-0.65)	5.54 (1.56-19.7)
WBC	≥10,300	74.0 %	38.9 %	71.1 %	42.4 %	0.57 (0.46-0.69)	1.90 (0.72-4.97)
CRP	≥7.4	64.4 %	69.4 %	81.0 %	49.0 %	0.65 (0.53-0.76)	3.35 (1.30-8.63)
Probable viral infection (*n* = 16)							
LIP2	≥896	87.5 %	28.7 %	17.3 %	93.1 %	0.50 (0.37-0.64)	3.50 (0.61-20.1)
SYN4^a^	≤5.64	81.2 %	37.2 %	18.1 %	92.1 %	0.54 (0.40-0.69)	7.16 (1.20-42.6)
WBC^a^	≤19,710	93.7 %	28.0 %	18.3 %	96.3 %	0.56 (0.42-0.70)	8.02 (0.75-85.1)
CRP^a^	≤5.2	75.0 %	64.5 %	26.7 %	93.7 %	0.67 (0.53-0.80)	5.95 (1.44-24.7)
Severe disease (*n* = 84)							
LIP2	≥783	83.3 %	50.0 %	84.3 %	48.1 %	0.64 (0.50-0.78)	5.45 (1.83-16.2)
SYN4^a^	≤5.83	71.4 %	50.0 %	82.2 %	35.1 %	0.58 (0.44-0.71)	4.37 (1.40-13.6)
WBC	≥11,170	73.5 %	69.2 %	88.4 %	45.0 %	0.71 (0.59-0.83)	8.34 (2.61-26.7)
CRP	≥4.56	67.5 %	61.5 %	84.8 %	37.2 %	0.65 (0.51-0.79)	2.72 (0.98-7.57)

*95 % CI* confidence interval, *CRP* C reactive protein, *LIP2* lipocalin-2, *OR* odds ratio, *SYN4* syndecan-4 (SYN4), *WBC* white blood cell count

Logistic regression model adjusting for age, sex, study center, ethnicity, parental education, and parental smoking habit

^a^Inverse relationship between biomarker and infection/severity of disease

CRP also remained the best predictor in the case of probable viral infection, although with marginal differences compared with the other studied parameters. The AUC for CRP was 0.67 (95 % CI 0.53-0.80) compared to 0.56 (95 % CI 0.42-0.70) for the WBC count, 0.50 (95 % CI 0.37-0.64) for LIP2, and 0.54 (95 % CI 0.40-0.69) for SYN4. All of the biomarkers had sensitivity but very low specificity, which led to an excellent NPV but low PPV.

The WBC count was the best predictor of severe CAP but differences among the studied variables were marginal. The AUC for the WBC count was 0.71 (95 % CI 0.59-0.83) compared to 0.65 (95 % CI 0.51-0.79) for CRP, 0.64 (95 % CI 0.50-0.78) for LIP2, and 0.58 (95 % CI 0.44-0.71) for SYN 4. LIP2 had the highest sensitivity of the cutoff values (83.3 %), with CRP having the lowest (67.5 %). The PPV values were 88.4 % for the WBC count, 84.8 % for CRP, 84.3 % for LIP2, and 82.2 % for SYN4. The NPV was low for all of the biomarkers.

All of the enrolled subjects showed significant improvement of their clinical conditions upon examination 5 days after enrollment. Table 4 summarizes the mean values of the studied biomarkers at 5 days after enrollment and the corresponding variations compared to the baseline data based on infection status. The WBC count was significantly lower on day 5 in children with probable bacterial CAP (*p* < 0.01) and in those with undetermined etiology (*p* < 0.01). CRP levels were significantly lower after 5 days from enrollment in all of the studied groups independent from the supposed etiology of CAP (*p* < 0.01 for all of the comparisons). The same was true for the LIP2 levels (*p* < 0.01). No statistically significant variations were observed for SYN4.

**Table 4:** Lipocalin-2 and syndecan-4 biomarkers at enrollment and corresponding variations after 5 days among 110 children hospitalized for radiologically confirmed community-acquired pneumonia according to their infection status

	All subjects	Probable bacterial £	Probable viral #	Undetermined
Biomarker	*n* = 110	*n* = 74	*n* = 16	*n* = 20
White blood cell at enrolment (cells/μL)	15,741 ± 8,575	16,573 ± 8,969	13,674 ± 7,338	14,358 ± 7,899
White blood cell after 5 days (cells/μL)^a^	9,623 ± 4,437	9,546 ± 4,817	10,396 ± 2,726	9,471 ± 3,942
Variation in white blood cell (cells/μL)^a^	−6,488 ± 9,680**	−7,505 ± 10,277**	−2,927 ± 8,310	−4,991 ± 7,899**
CRP at enrolment, μg/dL	25.5 ± 47.4	32.2 ± 55.5^***^	9.4 ± 17.0^***^	14.0 ± 19.4
CRP after 5 days, μg/dL^a^	6.8 ± 17.4	9.4 ± 20.7^***^	1.2 ± 1.9^***^	1.7 ± 3.3
Variation in CRP, μg/dL^a^	−20.2 ± 43.5**	−25.0 ± 51.4**	−6.6 ± 14.1**	−12.3 ± 16.7**
LIP2 at enrolment	2,079 ± 1,606	2,158 ± 1,744	1,880 ± 1,015	1,946 ± 1,488
LIP2 after 5 days	1,163 ± 1,015	1,195 ± 1,102	1,236 ± 828	986 ± 814
Variation in LIP2	−915 ± 1526**	−962 ± 1666**	−644 ± 583**	−959 ± 1532*
SYN4 at enrolment	5.08 ± 3.95	5.27 ± 4.10	4.31 ± 3.06	4.99 ± 4.14
SYN4 after 5 days	5.68 ± 4.29	5.71 ± 4.56	4.48 ± 3.08	6.53 ± 4.06
Variation in SYN4	0.59 ± 2.81	0.43 ± 2.75	0.17 ± 2.54	1.54 ± 3.17*

*CRP* C reactive protein, *LIP2* lipocalin-2, *OR* odds ratio, *SD* standard deviation, *SYN4* syndecan-4, *WBC* white blood cell count

*p < 0.05 between enrollment and day 5

**p < 0.01 between enrollment and day 5

^***^ *p* < 0.05 between probable bacterial and probable viral infection groups. No other statistically significant differences between the groups were found

^a^Data at day 5 regarding white blood cells were available for 92 subjects and CRP were available for 94 subjects

## Discussion

CAP is a significant clinical problem in children despite the availability of effective antibiotics and vaccines [3, 6]. Early identification of CAP cases due to bacterial infection or that are likely to become severe is critical to ensure appropriate treatment. Unfortunately, this study shows that in the studied pediatric population, neither LIP2 nor SYN4 were superior to CRP and the WBC count in predicting the etiology and severity of CAP and in the evaluation of disease course. Upon enrollment, the mean serum values of both LIP2 and SYN4 were similar in children with probable bacterial and probable viral infections. Moreover, the cut-offs with the highest sensitivity and specificity were either marginally or ineffective in identifying the etiology and predicting the severity. Although with suboptimal efficiency, CRP was associated with the highest efficiency in identifying bacterial and viral cases, and the WBC count was the most efficient biomarker in defining disease severity. Moreover, 5 days after diagnosis, when all of the enrolled subjects had shown significant clinical improvement, LIP2 had a reduction substantially similar to that observed in CRP and the WBC count, whereas SYN4 was insignificantly reduced. These findings do not support the use of these biomarkers in the routine clinical practice for identifying the probable etiology of a CAP case, defining its severity and evaluating its course. CRP and the WBC count provide slightly better information (particularly CRP), are included in laboratory tests routinely performed by all the hospital laboratories and are less expensive than testing for LIP2 and SYN4.

These conclusions are different from those reported by other authors. Nikaido et al. conducted a mass spectrometry-based proteomic study to identify and validate markers of bacterial etiology and severity of CAP in African children and reported that LIP2 could be effective for the identification of bacterial and severe cases [12]. However, the study population and methods used to evaluate the biomarkers were different, which could explain the contradictory results. In this study, otherwise healthy children hospitalized for CAP were enrolled, whereas Huang et al. evaluated subjects that in many cases were suffering from malaria, acquired immunodeficiency infection and malnutrition; all conditions that, as stated by the authors themselves, can significantly modify the LIP2 response [10]. Moreover, in our study, the criteria used to define probable bacterial and probable viral CAP and those used to define severe disease were more stringent. Data regarding the detection of respiratory bacteria and viruses in nasopharyngeal samples were associated with evaluated radiological findings according to the World Health Organization standardizations [20], whereas severity was established using the criteria suggested by the British Thoracic Society [21]. The results of this study are also different from those reported by Yeh et al. in adults in whom LIP2 was shown to predict the severity of CAP [9]. However, in this study, the criteria used to define severity were again different because pediatric criteria for the evaluation of CAP severity are not the ones used in adults.

Regarding SYN4, the data collected by Nikaido et al. in a small number of adults with CAP have shown that an increase in this biomarker could be observed only in subjects with mild disease, whereas its concentrations were similar to those found in healthy subjects when measured in patients with severe CAP [12]. Moreover, the serum levels of SYN4 were increased in patients who received antibiotics for a short period of time but not in those with long-term treatment [12]. That study was retrospective, and the criteria used for defining CAP severity were not stringent. Consequently, no comparison with the data collected in our study is possible.

In our study, serum CRP levels were the best predictor of etiology and WBC count the best predictor of severity. The potential use of CRP in differentiating bacterial from non-bacterial lower respiratory tract infection has been repeatedly suggested [28–31]. Similarly, WBC count is commonly used to identify among children with fever without source those who need antibiotic treatment because they are at risk of severe invasive bacterial infection [32]. However, none of these biomarkers can be considered the best solution for guiding clinical management of CAP in infants and children. CRP, as a type of acute phase reaction protein, is closely related to the inflammatory reaction and tissue injuries and can be significantly influenced by factors different from bacterial components [33]. For many years, it has been shown that only extremely high CRP serum levels are associated with bacterial disease and negative prognosis, whereas in many cases, these values do not allow the real etiology of the disease to be determined [28–31]. Similar conclusions are valid for the WBC count [28–31]. Among the attempts made in recent years to solve the problem of a prompt and effective supposition of the probable etiology of CAP, those involving procalcitonin evaluation appear to be the most reliable in suggesting etiology [34, 35]. Moreover, variations in procalcitonin concentrations can be used to establish a therapy response and antibiotic administration schedule [36]. However, in this study we did not evaluate procalcitonin because it was not available in routine practice in our hospital.

Limitations of this study include the fact that bacterial and viral detection in the upper respiratory tract could theoretically be positive also in asymptomatic carriers, only hospitalized children were evaluated, the study had descriptive purposes and the number of probable viral infections was quite low. However, the distribution of etiology seems to reflect results already obtained in hospitalized children [25, 27, 28] and the fact that we tried to differentiate bacterial and viral infections is a major strength of this research because previous studies were only focused on the use of the biomarkers in bacterial infection.

## Conclusions

This study indicates that the use of routine laboratory testing of LIP2 and SYN4 does not appear to be capable of defining the etiology of CAP in children. However, before definitively excluding the use of these biomarkers, further studies are needed. In lieu of a definitive answer, CRP and the WBC count, combined with adequate evaluation of clinical data, must be used in the attempt to limit the irrational use of antibiotics as much as possible and concurrently ensure the best treatment for the patient.

## Abbreviations

AUC, area under receiver operating characteristic curve; CAP, community-acquired pneumonia; CI, confidence interval; CRP, C reactive protein; ELISA, enzyme-linked immunosorbent assay; LIP2, lipocalin-2; NGAL, neutrophil gelatinase-associated lipocalin; NPV, negative predictive value; OR: odds ratio; PCR, polymerase chain reaction; PPV, positive predictive value; ROC, receiver operating characteristic; RSV, respiratory syncytial virus; RV, rhinovirus; SD: standard deviation; SYN4, syndecan-4; WBC, white blood cell
